# Ca analysis: An Excel based program for the analysis of intracellular calcium transients including multiple, simultaneous regression analysis^☆^

**DOI:** 10.1016/j.cmpb.2013.09.004

**Published:** 2014-01

**Authors:** David J. Greensmith

**Affiliations:** Unit of Cardiac Physiology, Institute of Cardiovascular Science, Manchester Academic Health Science Centre, Core Technology Facility, 46 Grafton Street, M13 9NT, UK

**Keywords:** Intracellular calcium, Calcium transient, Fluorescent indicators, Regression analysis, Visual basic for applications, Excel

## Abstract

Here I present an Excel based program for the analysis of intracellular Ca transients recorded using fluorescent indicators. The program can perform all the necessary steps which convert recorded raw voltage changes into meaningful physiological information. The program performs two fundamental processes. (1) It can prepare the raw signal by several methods. (2) It can then be used to analyze the prepared data to provide information such as absolute intracellular Ca levels. Also, the rates of change of Ca can be measured using multiple, simultaneous regression analysis. I demonstrate that this program performs equally well as commercially available software, but has numerous advantages, namely creating a simplified, self-contained analysis workflow.

## 1 Introduction

It is now commonplace to record and store experimental data in digital form. These data must then be analyzed. My own work involves the study of changes of intracellular calcium by using fluorescent Ca indicators [1,2]. The apparatus used provides the user with raw data in the form of voltage changes that reflect changes in intracellular Ca. However, this raw signal must be processed to provide meaningful physiological information. It must be prepared by one of several methods to provide acceptable ways of representing changes in intracellular Ca and only then can the prepared data be analyzed to quantify physiological phenomenon. These phenomena are varied, but include quantifying absolute levels of Ca (diastolic and systolic) and also measuring the rate of change of Ca.

Practically speaking, converting raw experimental data into meaningful physiological information requires several steps. Propriety software currently exists, such as ClampFit (Molecular devices LLC), Excel (Microsoft Corporation) and SigmaPlot (Systat Software Inc.) which can adequately perform some of these steps, but no single one can effectively complete the entire task making the process disjointed and time consuming. To improve the efficiency of this process, I have written an Excel based program. Excel is commonly used as a simple spread sheet and graphing package. However, by using Visual Basic for Applications (VBA), one can write macros that unlock Excel as a powerful tool for complex data manipulation. Arrange these macros into an efficient workflow and provide a user interface, and one essentially creates a self-contained program, using Excel as a chassis, where no user interface with Excel itself is required. Others have expanded Excel's capabilities in numerous ways, including using advanced features [3–5] creating bespoke add-ins [6,7], modeling biological systems [8–10] and writing self-contained programs [11].

Perhaps the greatest advantage of this approach is the fact that the program is essentially an Excel workbook containing all the necessary subroutines for the process. This means the user can, for a given bout of analysis, (such as recordings from a particular cell), save a copy of that workbook. Therefore, for a given cell, the raw data, prepared data, analysis process and results of analysis are self-contained and stored in one location. Subsequent and re-analysis can be performed by simply re-opening that workbook, negating the need to re-import raw data and re-export the result of preparation/analysis.

There are several other advantages. (1) Raw data can be imported from any source that allows an array of values to be copied to a PCs clipboard, such as Clampfit's basic data export routine. This negates the need to deal with unique, and program specific data file formats, which are at the mercy of alteration by version updates. (2) VBA is highly flexible allowing user specific modifications to be made to the core script without the need for re-compilation. (3) Compatibility issues are minimized as any PC capable of running Excel will run an Excel based program. (4) The financial benefits can be significant as a single piece of widely available software; Excel, can be used to create any number of bespoke software applications.

With regard to this program, for a given experiment, the program can hold the raw and prepared data of 10 specimen Ca transients. The absolute Ca levels described above can be measured and stored for each. In addition, for each specimen, the decay phase can be subjected to multiple simultaneous regression analysis. Although I discuss in terms of intracellular Ca analysis, any biological signal that requires analysis in this way can be processed by this program by simply bypassing the calibration steps.

## 2 Computational methods and theory

The program has 2 fundamental processing components. (1) Preparation of raw data, and (2) analysis of those prepared data. With regard to the latter, there are two sub components. (A) The analysis of absolute Ca levels and (B) multi-exponential fitting of the decay phase of the intracellular Ca transient in order to analyze kinetics.

### 2.1 Subtraction of background fluorescence during raw data preparation

Experimentally, photons are collected from the entire field of view “seen” by fluorophore imaging apparatus. Therefore “raw” florescence is that of the entire field of view. It is therefore standard practice to subtract background fluorescence (here, we regard background fluorescence to be that of the field of view with the cell removed). Irrespective of how background fluorescence is determined by a particular investigator, it must be subtracted from that of the raw fluorescence. This program automatically performs this step in all the raw data preparation algorithms (see Section 3 for practical aspects). Therefore, in all the equations given in the following sections, *F* is fluorescence subsequent to the subtraction of background fluorescence.

### 2.2 Preparation of data from ratiometric indicators

Dual excitation and dual emission fluorophores provide raw data in the form of a nominator and a denominator signal that must be ratioed prior to analysis (For example see [12]). This program automatically process the raw data as per Eq. (1).

(1) $$\text{Ratio}=\frac{F_{nom}}{F_{denom}}$$

where *F*_*nom*_ is the nominator fluorescent signal and *F*_*denom*_ is the denominator fluorescent signal.

### 2.3 Preparation of data from non-ratiometric indicators

The program can prepare the raw signal generated by non-ratiometric indicators in two ways. Calibration to a true intracellular Ca concentration (for example see [1]) is possible by implementation of Eq. (2) [13].

(2) $$[\text{Ca}]=\frac{FxKd}{F_{\max}-F}$$

where *F* is fluorescence (see above), *Kd* is the fluorophore's dissociation constant, and *F*_*max*_ is the fluorophore's maximal fluorescence.

Alternatively, raw data can be converted into a pseudo ratio (*F*/*F*_0_) (for example see [14]) where all Ca transients are normalized to a given, or their own resting fluorescent value by implementation of Eq. (3).

(3) $$F/F_{0}=\frac{F}{F_{0}}$$

where *F* is fluorescence and *F*_0_ is fluorescence in diastole.

### 2.4 Calculation of absolute Ca levels

Values of diastolic and peak systolic Ca are determined using cursors (see Section 3.3). The diastolic Ca value is subtracted from the peak value to calculate the Ca transient amplitude. If calibrated data are used, the result of this analysis gives absolute concentrations of Ca. If raw data prepared as *F*/*F*_0_ or ratios are used, this analysis gives relative signal changes that *reflect* changes of Ca.

### 2.5 Non-linear regression

The program determines the rate constant of decay of the experimental data (*Y*) by regression analysis using least square convergence. First, an initial predicted fit is generated by basic analysis of the experimental curve. The program then improves the fit of this predicted curve using an iterative loop implemented by the Excel Add-In “Solver” [4,15,16]. During each iteration, the predicted curves parameters are adjusted so generating a new predicted curve. Following each iteration, the goodness of fit is evaluated by summing the squares of the differences between the predicted and observed values. The predicted curve that results in the lowest sum of squares is the best fit. If the fit is robust, then the parameters of the observed curve can be inferred from those of the predicted.

#### 2.5.1 Fitting a single exponential decay to the experimental data

The predicted curve resulting from each iteration is generated by implementation of Eq. (4).

(4) $$Y_{predicted}=Y_{0}+(A\times \exp^{-kt})$$

where *Y*_*predicted*_ is the predicted value, *Y*_0_ and *A* are the baseline and amplitude of the observed curve (*Y*) respectively and *k* is the rate constant of decay of *Y*. To prevent excessive run durations, Solver may only use a finite number of iterations. Therefore, the “first guess” parameters must be relatively close to those of the best fit. The first guess values for *Y*_0_ and *A* are determined using cursors on a graphical user interface (Section 3.4). The initial value for *Y*_0_ is that derived from the “baseline” cursor's *Y*-axis position and the initial value of *A* is calculated from subtraction of initial *Y*_0_ from the value derived by the “peak” cursor's *Y*-axis position. The initial rate constant (*k*) is predicted from the experimental curves time constant (*τ*) as per Eq. (5).

(5) $$k=\frac{1}{\tau }$$

#### 2.5.2 Fitting a double exponential decay to experimental data

The predicted curve resulting from each iteration is generated by implementation of Eq. (6).

(6) $$Y_{predicted}=Y_{0}+(A_{1}\times \exp^{k_{1}t})+(A_{2}\times \exp^{k_{2}t})$$

where *Y*_*predicted*_ is the predicted value, *Y*_0_ is the curves baseline, *A*_1_ and *A*_2_ are the amplitudes of the two exponentials and *k*_1_ and *k*_2_ are the rate constants of decay of the two exponentials. For the initial prediction, *Y*_0_ is calculated as per Section 2.5.1. *A*_1_ and *A*_2_ are calculated as per Eq. (7a and 7b):

(7a) $$A_{1}=\frac{A}{a}$$

and

(7b) $$A_{2}=\frac{A}{b}$$

where *A* is the observed curves amplitude calculated as per Section 2.5.1, and *a* and *b* are user defined constants.

The initial guesses for the rate constants *k*_1_ and *k*_2_ are calculated as per Eq. (8a and 8b).

(8a) $$k_{1}=k_{c}$$

and

(8b) $$k_{2}=\frac{k}{d}$$

where *k* is the single exponential rate constant calculated as per Section 2.5.1, and *c* and *d* are user defined constants.

#### 2.5.3 Goodness of fit

The program allows the user to evaluate goodness of fit qualitatively by visual assessment of the overlaid predicted and observed curve (see Section 3.4). For a quantitative assessment, the program calculates the *R*^2^ value of the fit as per Eq. (9).

(9) $$R^{2}=1-\frac{SS_{reg}}{SS_{total}}$$

where *SS*_*reg*_ is the sum of the squares of the regression residuals (the difference of each *Y* data point and each *Y*_*predicted*_ data point), and *SS*_*total*_ is the sum of squares of the differences between each *Y* data point and the mean of *Y*.

To make *R*^2^ of the single and double exponential fit directly comparable, and therefore determine which describes *Y* most reliably, *R*^2^ is adjusted as per Eq. (10).

(10) $$\text{Adjustted}\;R^{2}=1-\frac{\left(SS_{reg}/(n-p)\right)}{\left(SS_{total}/(n-1)\right)}$$

where *n* is the number of data points and *p* is the number of parameters used to determine *Y*_*predicted*_. For most experimentally recorded curves, *n* will be in the order of 10^3^ and so *R*^2^ will not differ significantly from *adjusted R*^2^.

#### 2.5.4 Addition of other regression analysis by code integration

To date, the user has the option to fit experimental data with a single, double or simultaneous single and double exponential. However, any number of regression analyses could be incorporated with ease.

## 3 Program description and sample runs

**Fig. 1:**
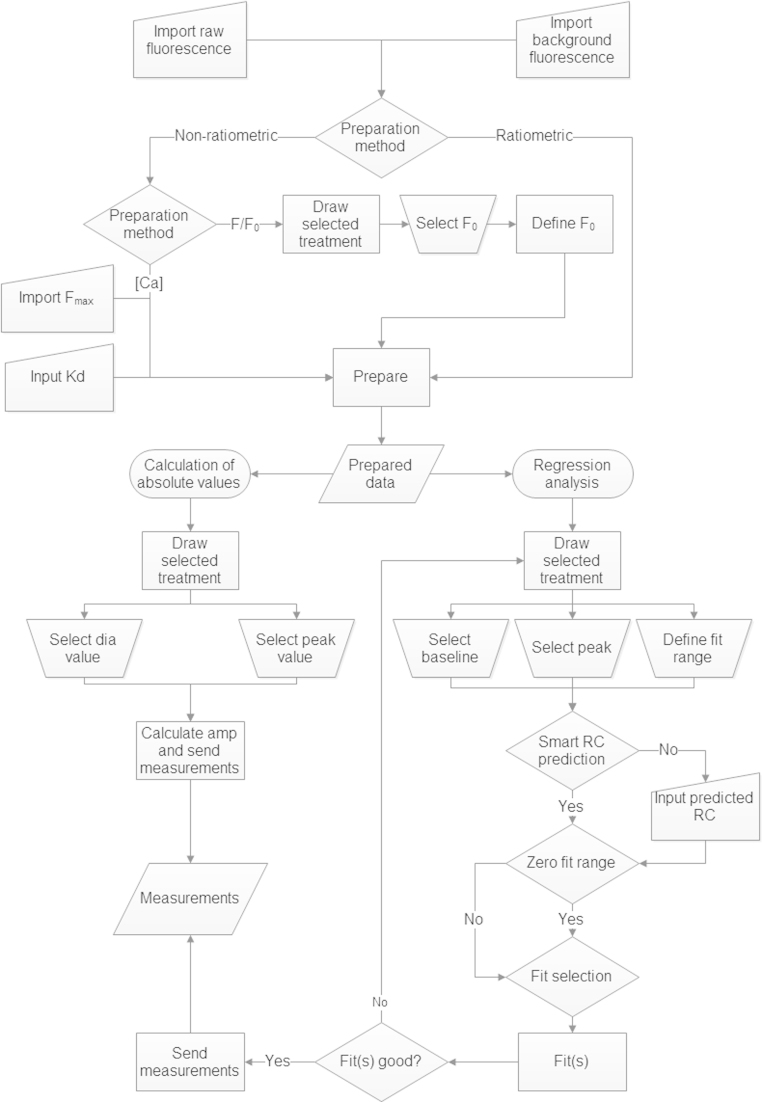
A flow diagram demonstrating workflow.

The program employs a logical workflow, taking the user through a series of steps, arranged as worksheet tabs. Each tab is responsible for a given step, and are ordered such that the user is taken from raw data input, to raw data processing, to analysis and finally to output. The workflow for a given step is logically arranged as a series of buttons, each of which triggering a particular sub-routine. A workflow overview is provided in Fig. 1.

We suggest that the program file is kept as a template, then, for a given experiment, the program is saved with a unique file name for a given cell or experiment. All the raw data, analysis and output for that experiment then becomes self-contained, and can be reopened for resumption of or re-analysis.

In the examples given in the following sections, specimen data were recorded from dog or rat cardiac ventricular myocytes loaded with either fura-2 or fluo-3.

### 3.1 Import of raw data

Two raw data sheets handle the import of raw data, one for ratiometric data, the other for non-ratiometric data. Here, I define a given specimen from an experiment as a treatment. Clicking a treatment's import button will bring data copied to the clipboard into the program.

### 3.2 Data preparation

**Fig. 2:**
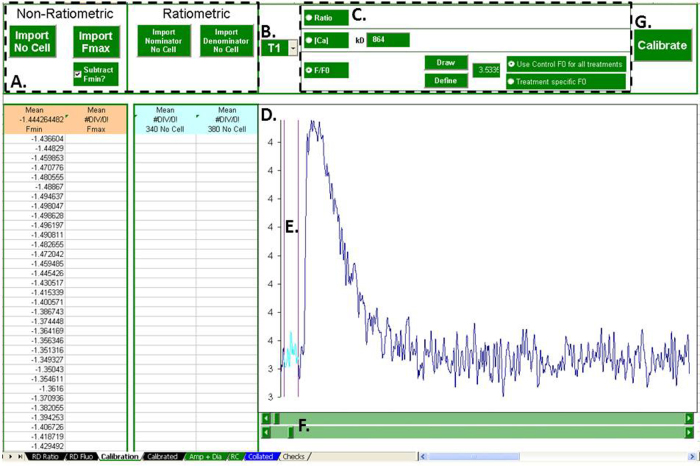
Raw data preparation. The “Calibration” tab deals with raw data preparation. The user must import the appropriate raw calibration data (A). The treatment for preparation is selected with a combo box (B). The user can then decide on the method of preparation (C). In the case of conversion to *F*/*F*_0_, *F*_0_ is defined from the plot (D) by flanking with the *X*-cursors (E) controlled with the scroll bars (F). The selected preparation can then be performed (G).

Preparation of non-ratiometric and ratiometric data is dealt with by the “Calibration” tab. (Fig. 2). First, the user must import the appropriate data for the preparation they wish to perform. All preparation types require background fluorescence data. Calibration of non-ratiometric data to [Ca] requires the indicators maximal fluorescence value (*F*_*max*_) and the fluorphores *Kd*.

The treatment to be prepared is selected with the combo box and the preparation type selected using the appropriate option button. To calculate ratios, or to calibrate to absolute calcium, the user just needs to click “Calibrate”. For conversion to *F*/*F*_0_, the user needs to define the value of *F*_0_ by selecting a treatment with the combo box then clicking “draw”. The currently selected treatment's Ca transient is then plotted, and the user flanks the region to be averaged as *F*_0_ using two cursors. Clicking define then averages that region, defining *F*_0_. Clicking “calibrate” then converts the currently selected treatment into *F*/*F*_0_. The user then has the option convert all the treatments with the current *F*_0_ or re-define *F*_0_ for each individual treatment.

The prepared data is then stored in the “Calibrated” tab for subsequent analysis by the program, or for export.

### 3.3 Calculation of absolute Ca levels

**Fig. 3:**
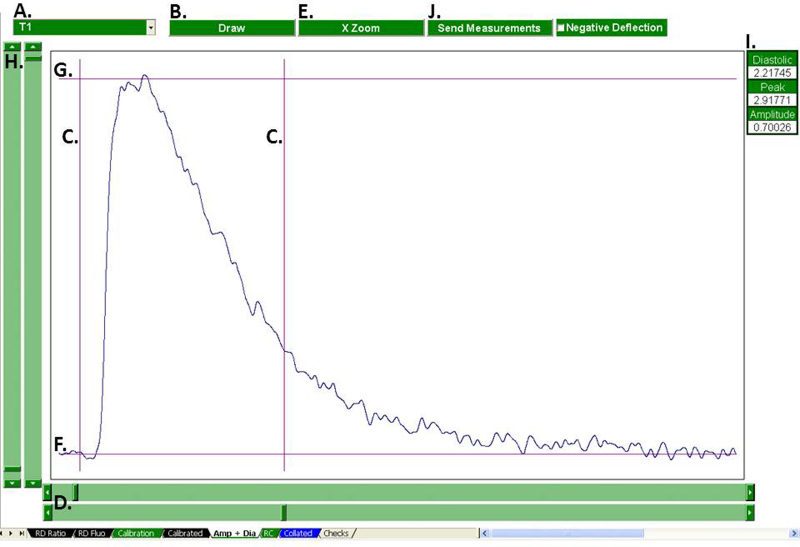
Calculation of absolute levels. Diastolic, peak and the Ca transient amplitude are calculated in the “Amp + Dia” tab. The treatment to be analyzed is selected with the combo box (A) and plotted (B). The *X*-cursors (C), controlled with *X*-scroll bars (D) can be used to flank a region of interest which can then be expanded (E). The diastolic (F) and peak (G) selection cursors are controlled with the *Y*-scroll bars (H). The current measurements are displayed (I) and can be sent to the “Collated sheet” once the user is content (J).

Calculation of diastolic, peak systolic and the calcium transient amplitude is performed by the “Amp + Dia” tab (Fig. 3). The user selects a treatment using the combo box and clicks “Draw”. The selected treatments Ca transient is then plotted, superimposed by a pair of *X*-axis cursors and a pair of *Y*-axis cursors. The position of these cursors is controlled using scroll bars. The user, if desired, can zoom to a region of interest by flanking that region with the *X*-axis cursors and clicking “Zoom”. The user defines the diastolic and peak Ca level by aligning the bottom *Y*-axis cursor with the diastolic region of the Ca transient and the top *Y*-axis cursor with the Ca transient's peak respectively. The amplitude is calculated automatically. Clicking “Send Measurements” sends the measurements to the “Collated” sheet.

### 3.4 Regression analysis

**Fig. 4:**
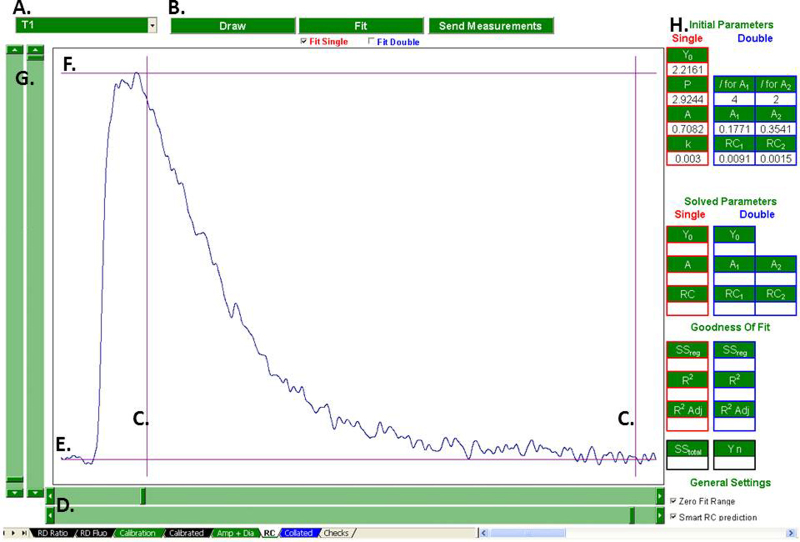
Preparations for regression analysis. Regression analysis of is performed in the “RC” tab but requires definition of both the fit region and initial predicted values. The treatment to be fitted is selected (A) and plotted (B). The fit region is defined using the *X*-axis cursors (C) controlled with the *X* scrollbars (D). The initial parameters for fit prediction are set by aligning *Y*-axis cursor E with the curves baseline and *Y*-axis cursor F with the curves peak, controlled with the *Y* scrollbars (G). The parameters for initial curve prediction are automatically calculated and displayed (H).

**Fig. 5:**
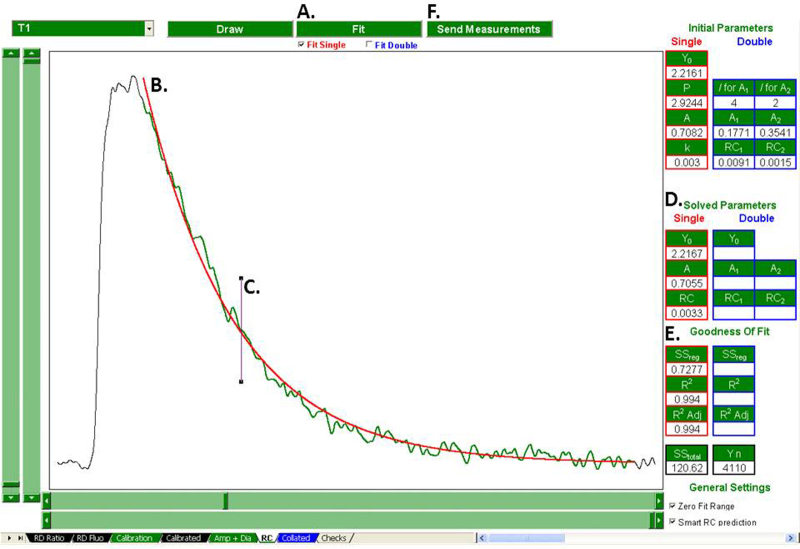
Visual assessment of goodness of fit. Once preparations have been made for regression analysis (Fig. 4), the selected region can be fit with a single and or double exponential. (A) This superimposes the predicted curve(s) onto the plot (B). If Smart RC Prediction is enabled, the location of Tau is also indicated (C). The solved parameters are displayed (D) as are the goodness of fit parameters (E). If the user is content with the fit, these parameters be sent to the “Collated” sheet (F).

Regression analysis, and so calculation of the rate constant of decay of the Ca transient is performed by the “RC” tab (Figs. 4 and 5). The user selects a treatment using the combo box and clicks “Draw”. The selected treatment's Ca transient is then plotted, superimposed by a pair of *X*-axis cursors and a pair of *Y*-axis cursors. Here, the *X*-axis cursors are used to flank the fit region. The user then aligns the bottom *Y*-axis cursor with the fit region's baseline, and the top *Y*-axis cursor with the fit region's peak. This provides the initial parameters for the iterative loop described in Section 2.5. The fit region can be zeroed (reducing the number of iterations required) by selecting the “Zero Fit Range” check box. Checking “Smart RC Prediction” enables the RC prediction algorithms outlined in Section 2.5.

The program is now ready to fit the selected region with a single exponential decay, a double exponential decay or both simultaneously, depending on the configuration of the fit check boxes. Clicking “Fit” fits the selected region with the appropriate regression(s) and the predicted curves are superimposed onto the plot so the user can evaluate the goodness of fit visually. If Smart RC Prediction is enabled, the position of tau is indicated with a vertical line. If the user is content with the fit(s), clicking “Send Measurements” sends the solved parameters to the “Collated” tab.

The goodness of fit parameters are also displayed (and exported upon click of “Send Measurements”.) These data give a quantitative assessment of the goodness of fit for the single and or double regression. Where both fits are performed simultaneously, they allow the user to quantitatively decide which regression describes the observed curve most reliably.

### 3.5 Output of analysis

**Fig. 6:**
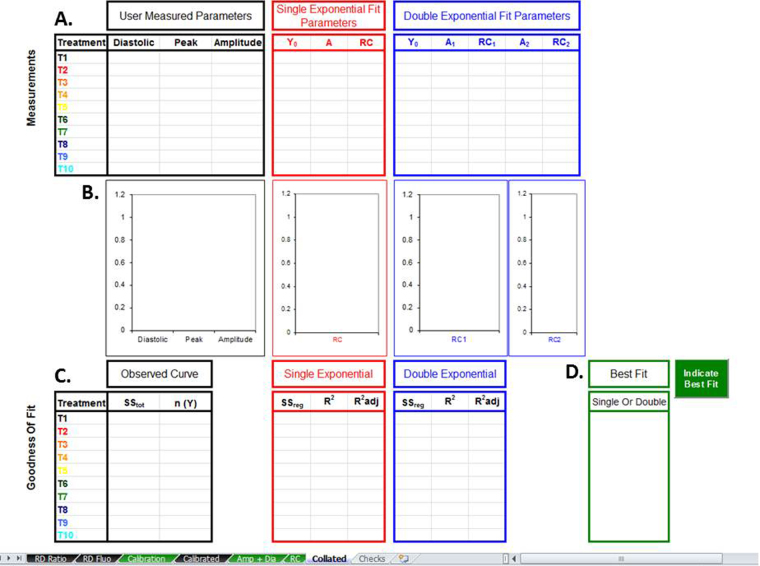
Collated data. All measurements are sent to the collated data sheet for convenient comparison or subsequent export. Collated data consist of absolute Ca values and fit parameters (A) and their respective histograms (B). A record of goodness of fit is provided (C). If simultaneous regression analysis was performed, that which describes the observed data most reliably can be indicated (D).

All measurements are sent to the “Collated” tab (Fig. 6). The data is arranged logically into tables, and automatically plotted as histograms. So long as the workbook is saved, the output of analysis will remain in the sheet. Or, the user can export as required.

If the user has opted to simultaneously fit the observed curve with a single and double exponential, clicking “Indicate Best Fit” gives an indication of which regression describes a given treatments data most reliably.

### 3.6 Accuracy of regression analysis

**Fig. 7:**
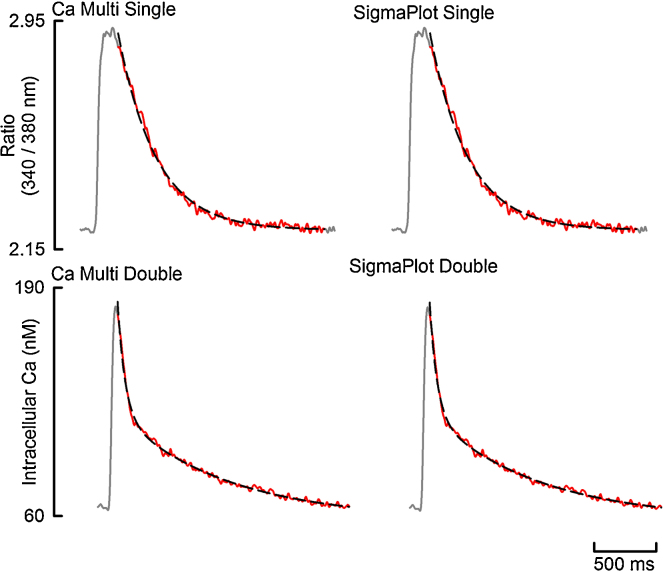
Validation of Fits. Top: single exponential fits. Bottom: Double exponential fits. In all figures, gray shows the entire Ca Transient, red shows the fit region and the black dash shows the fit. (For interpretation of the references to color in this figure legend, the reader is referred to the web version of the article.)

To test the reliability of the fit algorithms I compared the fit parameters derived using my program with those derived from the commercially available SigmaPlot. Each program was used to fit a single exponential to the decay phase of an example Ca transient, know to have a single exponential decay. I repeated this process, fitting a double exponential to an example Ca transient, know to have double exponential decay. The fit region compared was identical. Fig. 7 and Table 1 display the results of one such comparison. This comparison demonstrates that my program performs regression analysis as well as SigmaPlot.

**Fig. 8:**
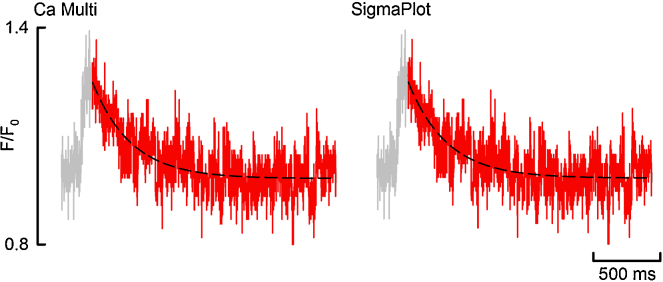
Fit performance with high noise. Gray shows the entire Ca Transient, red shows the fit region and the black dash shows the fit. (For interpretation of the references to color in this figure legend, the reader is referred to the web version of the article.)

The examples given above are from relatively clean signals. Regression reliability can decrease as the variance (*SS*_*total*_) of *Y* increases due to, for example, noise. To test how reliably this program would deal with such a scenario, a calcium transient with a relatively poor signal to noise ratio was fitted with a single exponential. Fig. 8 and Table 2 display the outcome of this fit. Fig. 8 visually demonstrates that the fit is robust and is comparable to that performed using SigmaPlot. Table 2 quantitatively confirms the fit is robust, and the program performs as well as SigmaPlot when fitting signals with a poor signal to noise ratio.

### 3.7 Comparison of simultaneous regression

**Fig. 9:**
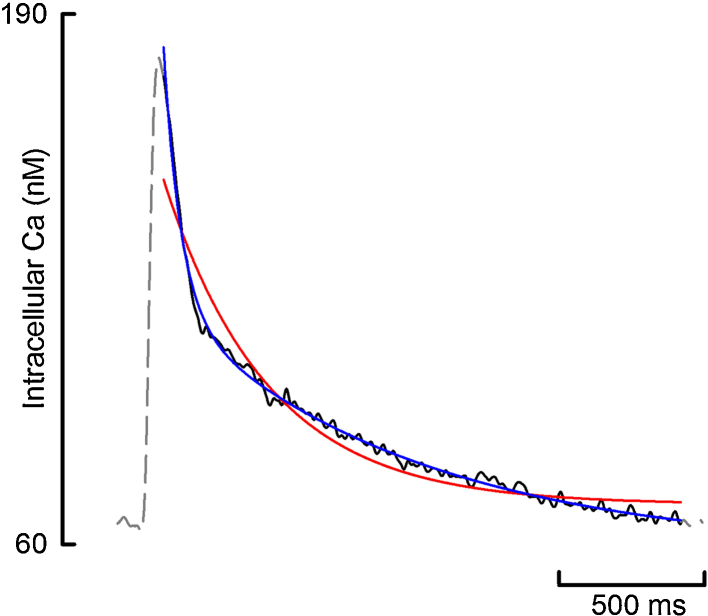
Comparison of simultaneous single and double exponential fit. Gray dash shows the entire Ca transient with the fit region in black. Red shows a single exponential fit and blue shows a double exponential fit. (For interpretation of the references to color in this figure legend, the reader is referred to the web version of the article.)

A useful feature of the program is the ability to simultaneously fit the same region with a single and double exponential so as to decide which describes the observed data most reliably. The goodness of fit parameters provide a quantitative assessment of which is best, and those from each fit are directly comparable due to the fit region being identical. Fig. 9 shows the result of an example simultaneous fit and Table 3 compares the goodness of fit parameters. It is clear visually that the double exponential is the better of the two fits. Comparison of the goodness of fit parameters in Table 3 confirms this.

## 4 Hardware and software specification

This program will run on any PC capable of running Microsoft Excel. It is compatible with Microsoft Excel 2003, 2007 and 2010.

## 5 Mode of availability

A copy of the program and instructions for initial set-up can be obtained by e-mailing the author.

## Figures and Tables

**Table 1:** A comparison of fit parameters derived using Ca analysis and SigmaPlot demonstrated in Fig. 7.

Parameter	Ca analysis	SigmaPlot
**Single exponential**		
*Y*_0_	2.217389	2.2174
*A*	0.688976	0.6890
*k*	0.003334	0.0033337

**Double exponential**		
*Y*_0_	57.96	58.11
*A*_1_	64.19	64.01
*k*_1_	0.017	0.017
*A*_2_	59.76	59.80
*k*_2_	0.0012	0.0012

**Table 2:** Fit reliability with noisy data demonstrated in Fig. 8.

Parameter	Ca analysis	SigmaPlot
**Single exponential**		
*Y*_0_	0.9823	0.9823
*A*	0.2654	0.2654
*k*	0.00355	0.00355

**Table 3:** A comparison of the goodness of fit parameters for the simultaneous fit demonstrated in Fig. 9.

Parameter	Single exponential fit	Double exponential fit
*SS*_reg_	79,588	6562.1
*R*^2^	0.9448	0.9954
